# Learning to suppress a location is configuration-dependent

**DOI:** 10.3758/s13414-023-02732-2

**Published:** 2023-05-31

**Authors:** Ya Gao, Jasper de Waard, Jan Theeuwes

**Affiliations:** 1 Department of Experimental and Applied Psychology, Vrije Universiteit Amsterdam, Van der Boechorststraat 7, 1081 BT Amsterdam, The Netherlands; 2 Institute Brain and Behavior Amsterdam (iBBA), Amsterdam, the Netherlands; 3 William James Center for Research, ISPA-Instituto Universitario, Lisbon, Portugal

**Keywords:** Visual search, Attentional capture

## Abstract

Where and what we attend is very much determined by what we have encountered in the past. Recent studies have shown that people learn to extract statistical regularities in the environment resulting in attentional suppression of locations that were likely to contain a distractor, effectively reducing the amount of attentional capture. Here, we asked whether this suppression effect due to statistical learning is dependent on the specific configuration within which it was learned. The current study employed the additional singleton paradigm using search arrays that had a configuration consisting of set sizes of either four or 10 items. Each configuration contained its own high probability distractor location. If learning would generalize across set size configurations, both high probability locations would be suppressed equally, regardless of set size. However, if learning to suppress is dependent on the configuration within which it was learned, one would expect only suppression of the high probability location that matched the configuration within which it was learned. The results show the latter, suggesting that implicitly learned suppression is configuration-dependent. Thus, we conclude that the high probability location is learned within the configuration context within which it is presented.

## Introduction

Although massive amounts of information are constantly bombarding our senses, we seem to effortlessly direct attention to relevant information and ignore information that may distract us. For a long time, attentional selection was thought of as the result of the interplay of top-down and bottom-up control processes (Corbetta & Shulman, 2002; Desimone & Duncan, 1995; Posner & Petersen, 1990), operating in a winner-takes-all way. However, a surge of recent studies has demonstrated that efficient selection to a large extent relies on what has been labelled as ‘selection history’. Selection history includes phenomena such as contextual cueing, reward and punishment associated learning, and probability cueing effects (Awh et al., 2012; Failing & Theeuwes, 2018), and these attentional biases cannot be explained by the observer’s current goals nor by the physical salience of the stimuli. For example, a task-irrelevant item previously associated with reward or punishment can automatically capture attention regardless of current goals or salience (Anderson et al., 2011; see Watson et al., 2019, for a review).

Visual statistical learning (VSL) of target and/or distractor locations has a large effect on attentional selection (see Theeuwes et al., 2022, for a review). VSL refers to the mechanism that enables observers to extract the distributional properties from sensory input across time and space (Frost et al., 2015). For example, so called contextual cueing studies demonstrated that search for a target is more efficient when it appears reliably in specific locations within displays previously searched relative to when these targets appear at unpredictable locations within new displays (Chun & Jiang, 1998). Also, Geng and Behrmann (2005) showed that targets presented in high-probability locations are detected faster than those in low-probability locations (see also Ferrante et al., 2018; Jiang et al., 2013).

These studies indicate that people easily pick up on statistical regularities concerning the location of the target. Recently however, a large number of studies demonstrated that not only target but also distractor-based regularities affect attention deployment (e.g., Failing & Theeuwes, 2020; Ferrante et al., 2018; Goschy et al., 2014; Wang & Theeuwes, 2018a, b, c; Feature-based: Vatterott & Vecera, 2012). Wang and Theeuwes used the additional singleton paradigm, and manipulated the spatial distribution of the color singleton distractor. Specifically, the color distractor was presented more often in one location relative to the other locations. Participants learned to suppress this high probability location as demonstrated by faster responses to the target when the distractor was presented at the high probability location relative to when it was presented at low probability locations. Moreover, participants responded slower when the target happened to be presented at this high probability distractor location (Wang & Theeuwes, 2018a, b, c; also, see Ferrante et al., 2018; Goschy et al., 2014). Following studies also suggested that this type of statistical learning occurs without much effort (Duncan & Theeuwes, 2020; Gao & Theeuwes, 2020), largely occurs outside awareness (Wang & Theeuwes, 2018b), and is not influenced by explicit knowledge of the regularity (Gao & Theeuwes, 2022).

However, at this point it is not immediately clear what exactly is learned during statistical learning. One possibility is that observers simply learn to inhibit a specific location on the display where the distractor is most likely to appear. The idea is that each time a distractor is presented at that location, this specific location gets suppressed regardless of the surrounding display elements (the display configuration). Alternatively, the suppression observed may depend very much on the surrounding context such that suppression is only applied when it fits the context within which it was learned. Up until now, all studies investigating attentional suppression have used display configurations that remained the same throughout the experiment, so that these alternatives cannot be teased apart.

Recent studies examined the relationship between the learned suppression effect and the global context in which it was learned. Britton and Anderson (2020) and de Waard et al. (2022) used the additional singleton task and presented this task with different backgrounds (i.e., natural scenes, brightness of background). Each background was associated with one specific high probability location in the search array. These experiments showed that statistically learned suppression was not context-dependent but instead generalized across the different contexts, suggesting that there was no learning of the association between the background and the specific high probability location within the search array. These findings are consistent with the idea that the distractor location becomes suppressed regardless of the background context within which learning took place. However, the question is whether in these kinds of experiments the background is strong enough to be associated with one specific location within the search array. Indeed, the background is often completely irrelevant for the task (for an exception, see Experiment 3 in de Waard et al., 2022) and suppressing both locations simultaneously appeared to incur little to no cost (i.e., participants are easily capable of this) as compared with suppressing one specific location in its corresponding context.

However, some previous studies have shown context dependent learning. Studies investigating reward and punishment associative learning have shown that contextual information influences attentional learning during visual search (Anderson, 2015; Grégoire et al., 2020). Counter to de Waard et al. (2022) and Britton and Anderson (2020), Allon and Leber (2019) provided evidence that spatially learned suppression can be implemented in a context-dependent fashion, in which participants could associate different backgrounds with different high-probable distractor locations. Furthermore, Turatto et al. (2018) showed that the reduction in attentional capture by irrelevant onsets relied on a stored representation in relation to their context.

Across the board, studies investigating the role of context in visual search (with the exception of contextual cueing studies) typically employed a context manipulation of the background, while the search display configuration remained the same. It is entirely possible that the actual display configuration in which search takes place is a much stronger ‘context’ than the background information. As participants acquire higher levels of configuration information, they may learn to rely more on the location within a particular display configuration than on the isolated location.

The different role of local configuration information and global properties of environment has been extensively studied in contextual cueing paradigms. Target search is facilitated as participants learn the spatial relationship between repeated search displays and target locations, so that the non-target items serve as an implicit ‘cue’ for the target location (Chun & Jiang, 1998). Contextual cueing studies have shown that the learned target location was more sensitive to the spatial configuration of the objects closest to the target (Brady & Chun, 2007; Olson & Chun, 2002). In fact, local variability around the target blocked contextual cuing altogether (Olson & Chun, 2002). On the other hand, Jiang and Wagner (2004) found that both individual target-distractor associations and overall display configurations are learned in contextual cuing. When Brooks et al. (2010) combined contextual cueing and background context into a single study, they observed that the contextual cueing effect became paired with the background information: changing the background disrupted the contextual cueing effect. All these results are consistent with the assumption that local configuration information could be critical for context-dependent learning to occur.

Given the discrepancies between context-dependent and independent suppression in the literature, the present study examined the role of configuration information in statistical learning of distractor suppression. More specifically, we asked whether the learned suppression effect is associated with the configuration context. We created two configurations, in which the spatial relationship of the search items was either static or variable. In the static condition, 10 search items (including one target, one distractor, and eight non-singleton stimuli) were placed equidistantly on an imaginary circle. The variable condition contained only four search items, because six of the eight non-singleton stimuli were randomly removed such that the layout of the search items varied from trial to trial. Furthermore, each configuration condition was paired with a different high probability distractor location. By comparing the distribution of suppression for these two conditions, the present study sought to determine if the configuration information was encoded during statistical learning. If this distractor-configuration association is learned, we should find a configuration-dependent suppression effect: only the one high-probability location of that configuration would be suppressed. Conversely, if we find that both locations are suppressed independent of configuration, this would suggest that suppression is learned independent from the context.

## Methods

### Participants

The critical comparison in the present studies is between trials with distractors appearing at a high-probability configuration-matching location versus configuration-mismatching location. Following de Waard et al. (2022), we adopt an effect size of 0.45, which means at least 54 participants were required to yield power over 0.90 with α set to 0.05 (using G*Power 3.1). Sixty participants (mean age = 25.25, *SD* = 3.3, 41 female, 15 male, 3 non-binary) were recruited online through Prolific (Palan & Schitter, 2018). Six participants were replaced because of low accuracy (<70%). The experiment lasted approximately 35 minutes and participants were compensated £4 for completing it. All participants had normal or corrected-to-normal vision and were naïve to the purpose of the experiment. Only participants between the ages of 18 to 35 years who acquired at least an undergraduate degree were allowed to participate. Informed consent was required before the start of the experiment. The ethical committee of the Faculty of Behavioral and Movement Sciences of Vrije Universiteit Amsterdam approved the study.

### Apparatus and stimuli

The experiment was created in OpenSesame (Mathôt et al., 2012) using OSweb, and run using JATOS (Lange et al., 2015). Because participants took part in this experiment online through their own computer or laptop, some factors (e.g., lighting and seating conditions) could not be controlled. The initial resolution of the experiment was set to 1024 x 768 pixels. All stimuli sizes and colors are reported in pixels and RGB values (red/green/blue).

**Fig. 1:**
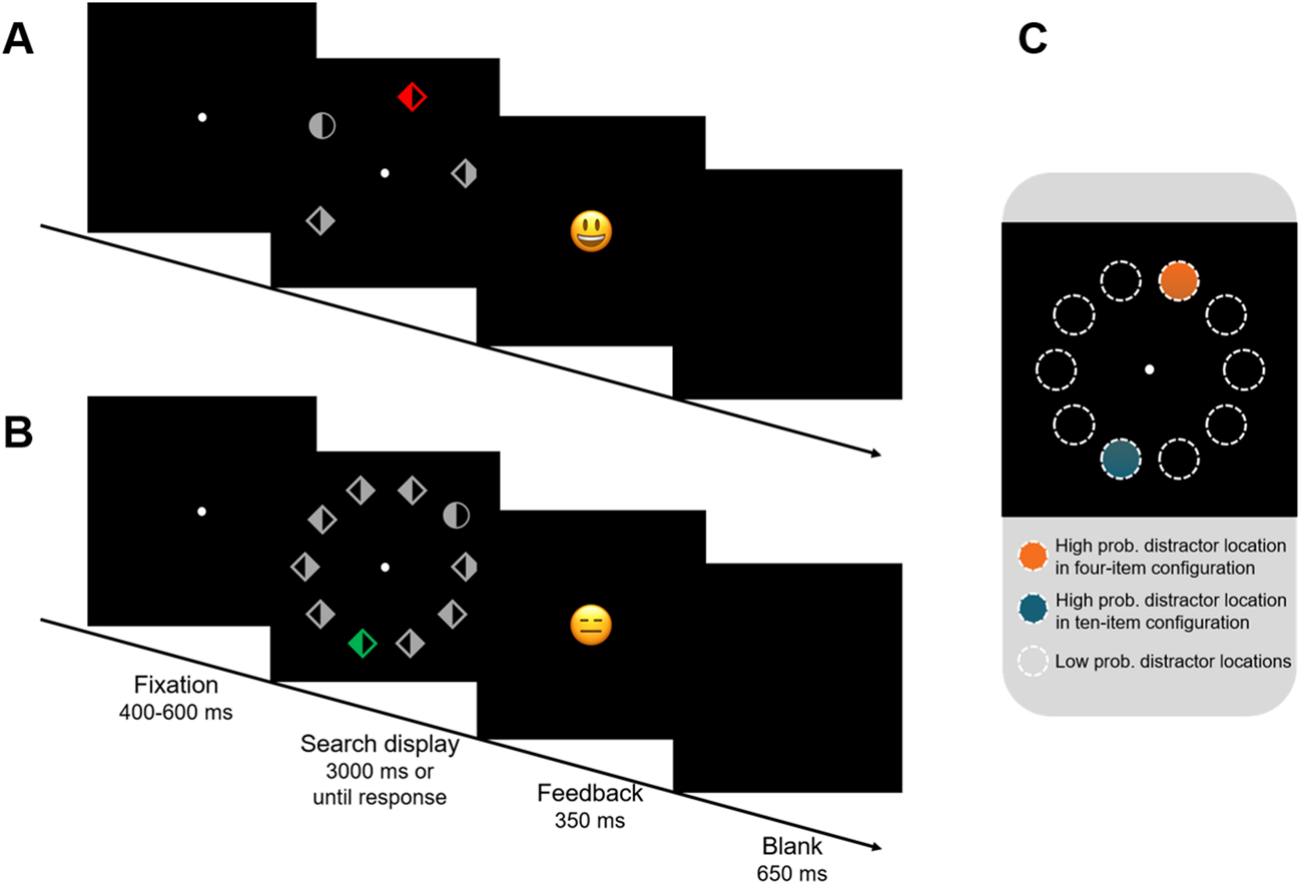
Time course of events for an example trial within **A** four-item configuration and **B** 10-item configuration. A white fixation dot was shown for 400 to 600 ms (randomly selected), followed by a search display which was visible until response with a maximum of 3 seconds. The task was to find the uniquely shaped item (target) and indicate as quickly as possible which of its sides was filled by pressing the left or right arrow key. A smiley (correct) or frowny (incorrect) provided feedback after every trial. The intertrial interval was 650 ms. **C** Schematic representation of spatial distribution of the distractor. The white dashed circles represented all possible search locations. Each configuration context had its own high probability distractor location (marked in orange and blue), and these two locations were always kept maximally distant for each participant. The rest of the locations were shown as low probability distractor locations. (Color figure online)

Some example displays are shown in Fig. 1. Two different search configurations of varied array sizes (ten or four items) were created to be paired with different high-probability locations. The 10 possible search locations were placed on an imaginary circle (radius 240 pixels) centered on fixation. For the 10-item array, nine circles and one diamond or vice versa were presented at these 10 locations, constituting the static configuration context. The four-item array showed three circles and one diamond or vice versa, constituting the variable configuration context. These configurations were variable in the sense that while the target (equally often at every location) and distractor (could be present/absent, high/low probability) locations were determined beforehand, the locations of the remaining items were chosen randomly from the 10 possible locations from trial to trial. All items were grey (128/128/128), except for the distractor which was red (255/102/102), green (102/255/102) or yellow (255/255/102). Every item (130 pixels in diameter) was horizontally split, with one half designed as an outline (12 pixels thick) and the other half filled. The background was black.

### Design and procedure

Figure 1 gives a schematic overview of a single trial under each configuration. A white fixation dot was shown for 400 to 600 ms (randomly selected), followed by a search display which was visible until response with a maximum of 3 seconds. The task was to find the uniquely shaped item (target) and indicate as quickly as possible which of its sides was filled by pressing the left or right arrow key. A smiley (correct) or frowny (incorrect) provided feedback after every trial. The intertrial interval was 650 ms.

A target was present on every trial, and a distractor was present on 75% of the trials (25% for each distractor color). Critically, the distractor occurred more often in one specific location (37.5%) than the other locations (4.17% per location), depending on the configuration context. The two high-probability locations (one for each configuration) were counterbalanced across participants, and remained constant throughout the experiment. The target was presented equally often at every location. The overall spatial distribution of the target and distractor were completely identical in the 10 and four-item arrays, except that the high probability location of static configuration context was always opposite to that of the variable configuration context.

Participants completed at least 30 practice trials, and would do 30 more if their accuracy did not exceed 60%. No spatial regularity of the distractor was involved in the practice phase. The formal experiment consisted of 480 trials in total, divided in blocks of 80. The two configuration contexts occurred equally often, and were randomly intermixed. Awareness of the spatial regularities was assessed after all trials were completed. Participants indicated one high probability location followed by a confidence rating for each configuration context.

## Results

For RT analysis, we excluded 5.1% error trials and 2.7% of trials with either an RT larger than 2.5 standard deviations from the average RT per condition per participant or an RT below 200 ms.

Repeated-measures analysis of variance (ANOVA) and paired *t* tests were performed to verify if the uneven distribution of distractor locations modulated attentional capture. In addition to conventional statistical analysis, Bayesian repeated measures ANOVAs (BF_incl_ across matched models; Wagenmakers et al. (2018)) and Bayesian paired *t* tests (BF_10_; the ratio of the likelihood of the alternative hypothesis H1 relative to the null hypothesis H0) were used to calculate the effect probability through JASP (JASP Team, 2022).

### Does statistical learning occur under both configurations?

**Fig. 2:**
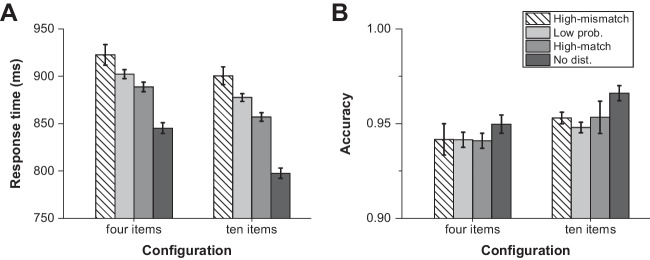
RT and accuracy data as a function of distractor condition (high-mismatch, low probability, high-match, or absent), separated for each configuration context. Here ‘high-match’ refers to the high probability location in its own configuration context; ‘high-mismatch’ refers to the high probability location in the other configuration context; ‘low probability’ refers to the remaining locations except for the high probability locations. Error bars indicate 95% within-subject confidence intervals (Cousineau, 2005). **A** Response times. **B** Accuracy

Figure 2 shows RT and accuracy data for the four distractor conditions (high-mismatch, low probability, high-match, or absent) and the two configuration contexts (four or 10 items). We performed a 4 (distractor condition) × 2 (configuration context) repeated-measures ANOVA for both the RT and accuracy data. The RTs showed a significant effect of *configuration*, *F*(1, 59) = 22.635, *p* < . 001, *η*_*p*_^*2*^ = .277, BF_incl_ > 1000, indicating that participants were relatively slower in the four-item configuration. A possible reason for this is that the target is less salient when there are fewer items on the display (Bravo & Nakayama, 1992; see also Theeuwes, 2023, for a discussion). The main effect of *distractor condition* was also significant, *F*(3, 177) = 62.555, *p* < . 001, *η*_*p*_^*2*^ = .515, BF_incl_ > 1000. Planned comparisons showed that the distractor captured attention reliably in the four-item configuration, *t*s(59) > 5.262, *p*s < .001, *d*s > .679, BF_10_ > 1000, and in the 10-item configuration, *t*s(59) > 9.905, *p*s < .001, *d*s > 1.279, BF_10_ > 1000. Furthermore, participants learned the distractor regularity in both configurations, as evidenced by faster RTs in the high-match compared with the low-probability distractor trials in the four-item configuration, *t*(59) = 2.131, *p* = .037, *d* = .275, BF_10_ = 1.15, and the 10-item configuration, *t*(59) = 3.741, *p* < .001, *d* = .483, BF_10_ = 58.99. There was no interaction between these two factors, *F*(3, 177) = 1.577, *p* = .215, *η*_*p*_^*2*^ = .026, BF_incl_ = .108.

The accuracy results only showed a main effect of *configuration, F*(1, 59) = 7.755, *p* = . 007, *η*_*p*_^*2*^ = .116, BF_incl_ = 5.487, that is participants made fewer errors in the 10-item configuration than that in the four-item configuration. Besides, no main effect of distractor condition, *F*(3, 177) = 2.297, *p* = .109, *η*_*p*_^*2*^ = .037, BF_incl_ = .15, or interaction, *F*(3, 177) = .241, *p* = .739, *η*_*p*_^*2*^ = .004, BF_incl_ = .027, was observed. These results indicate that the RT results cannot be ascribed to speed–accuracy trade-offs.

### Is the learned distractor suppression configuration‑dependent?

As shown in Fig. 2A, we directly compared the RTs of high probability configuration-matching trials with high probability configuration-mismatching trials. If the learned suppression in one configuration transferred to another configuration, we should find that participants responded equally fast for high-mismatch as for high-match trials. By contrast, if the suppression was learned in a configuration-dependent way, the high-match condition should yield faster RTs than the high-mismatch condition. Paired *t* tests revealed that indeed participants were faster in the high-match compared with the high-mismatch condition in the four-item configuration, *t*(59) = 2.764, *p* = .008, *d* = .267, BF_10_ = 4.445, and in 10-item configuration, *t*(59) = 4.368, *p* < .001, *d* = .267, BF_10_ > 100. These results indicate that learning of the distractor location regularities was configuration-dependent.

Compared with the low probability condition, RTs of the high-mismatch were slower in 10-item configuration, *t*(59) = 2.064, *p* = .043, *d* = .267, BF_10_ = 1.017, and showed no difference in four-item configuration, *t*(59)= 1.649, *p* = .104, *d* = .213, BF_10_ = .505. In both configurations, the distractor interfered the most with search in the high-mismatch condition, indicating that the high-mismatch locations were not suppressed at all. The slow response times in the high-mismatch condition could be due to the stimuli being farthest from the high probability distractor location within its own context, given the opposite positioning of the two high probability distractor locations.

### Awareness of the regularities

Sixteen participants reported that they noticed some regularities concerning the distractor location. Overall, participants were not confident about their answers (mean confidence rating of 2.0 on a Likert scale of 1 to 5), but the aware group (Mean Likert = 2.78) showed more confidence than the unware group (Mean = 1.73) in both configurations, *t*s(58) > 3.093, *p*s < .003, *d*s > .903, BF_10_ > 12. Crucially, however, it appears that this confidence was unjustified. We calculated the distance between the locations indicated by participants and the actual high-probability location for each configuration, and compared those distances between aware and unaware groups by conducting a Bayesian independent *t* test. No significant difference was observed in the four-item configuration, *t*(58) = .049, *p* = .961, *d* = .014, BF_10_ = .29, or in the 10-item configuration, *t*(58) = .613, *p* = .542, *d* = .179, BF_10_ = .343, indicating that participants were guessing. If we excluded the sixteen ‘aware’ participants, the difference in RTs between the high-match and high-mismatch conditions was still significant in the 10-item configuration, *t*(43)= 3.497, *p* = .001, *d* = .527, BF_10_ = 27.12, but not in the four-item configuration, *t*(43)= 1.929, *p* = .06, *d* = .291, BF_10_ = .886, which could be due to the reduced sample size.

## Discussion

The present study provides evidence that, at least in the conditions tested here, learning to suppress a distractor location is configuration specific, given that there was only suppression of the location that matched the configuration within which it was learned. Using the additional singleton paradigm, we created two configuration contexts (four-item or 10-item search displays), and assigned each configuration context its own high probability distractor location. This design allowed us to investigate whether participants learned to suppress each location exclusively within its respective configuration, or alternatively whether learning generalized across configurations such that both high probability locations would be suppressed equally irrespective of the context. The data are clear in that participants only learned to suppress the high probability location for the configuration within which it was learned, providing strong evidence for configuration-dependent learning.

The current findings indicate that observers do not simply learn to inhibit a specific location where the distractor is most likely to appear. If this would be the case one would expect that irrespective of the configuration within which the distractor was presented, attentional capture of the distractor would be attenuated. The results clearly show the opposite as attenuation of capture was configuration dependent. Related to this notion, the current findings may at first appear to be inconsistent with the view that suppression is the result of what has been labelled as ‘habituation’ (e.g., Pascucci & Turatto, 2015; Won & Geng, 2020). Habituation is considered a form of non-associative learning whereby a decrement in response is observed when a stimulus is repeated (Groves & Thompson, 1970). Therefore, attentional suppression as observed here could simply be the result of a repeated presentation of a stimulus at a particular location. According to this view of habituation, one would expect then that there should be suppression of both high probability locations independent of context, as the stimulus was presented at both locations repeatedly. However, Wagner (1979) suggested an associative theory of habituation, claiming that through training an association is formed between the repetitive stimulus and its surrounding context. Only when exposed to the same context, the habituated stimulus representation is retrieved for short-term memory (Turatto & Pascucci, 2016). As mentioned earlier, in relation to attentional capture, Turatto et al. (2018) provided compelling evidence for this associative theory of habituation. They showed that habituation of attentional capture by irrelevant onsets relied on a stored representation in relation to their context. The current findings are consistent with this view showing clear evidence that the reduction in attentional capture is context dependent.

The question is then why the current experiment (and that of Turatto et al., 2018) show a clear context effect while previous studies (Britton & Anderson, 2020; de Waard et al., 2022) found no evidence for context-specific learning. The answer to this question may be related to the way context was manipulated. In Britton and Anderson (2020) and de Waard et al.’s (2022) studies, context was manipulated by presenting the search array on top of different backgrounds. The results showed that learned suppression was independent of the background context, as high probability locations were suppressed equally strong regardless of the background. In the current study and that of Turatto et al. (2018), context was manipulated by different search display layouts. In our study, there were either four or 10 search items on display and each representing a different context. The statistical learning (SL) of distractor happened separately and independently within its own configuration context, which was consistent with the notion that implicit, rather than explicit, knowledge is often relatively inflexible in transfer to different domains (Dienes & Berry, 1997). Turatto et al. (2018) showed participants that were previously trained with displays that no longer contained an onset distractor, showed full recovery of attentional capture when the onset distractor was reintroduced suggesting that habituation of capture was context specific.

The current context effects appear to be related to findings related to contextual cueing (Brady & Chun, 2007; Jiang & Wagner, 2004; Olson & Chun, 2002). In these studies, participants learn target locations in relation to particular configuration contexts. While related, the current context effects are quite different from those employed in contextual cueing. For example, in our four-item displays the specific display layout (i.e., where items are presented on the screen) varies dramatically between trials, which is unlike the specific display layout learning that takes place in contextual cueing. For example, Olson and Chun (2002) found no evidence for contextual cueing when the sub-region around the target was varied, despite the invariability in other regions. This indicates that learning that takes place in contextual cueing studies is quite different from the learning in the current study.

Finally, it should be noted that in the current study the different configuration contexts might have induced different search strategies. In this respect, the context specific suppression of the high probability location may be associated with the way the search tasks are performed. It is feasible that the four-item display induced a more serial (clump-wise) search mode because the target was less salient (Wang & Theeuwes, 2020). This claim is consistent with the observed search times: even though within the four item displays there were much fewer display elements to inspect, search times were significantly slower than in the 10-item display. These findings are consistent with the notion that search was (partly) serial within the four-item display and parallel in the 10-item display (see also Theeuwes, 2004, for a similar argument).

In sum, the current study provides evidence for context-specific learned suppression. The absence of any transfer between different display configuration contexts suggests that it is possible to create conditions that allow configuration dependent, implicitly learned suppression.

## Open practices statement

Data for the experiment are available online (https://github.com/YaGao28/configurationSpecific), and none of the experiment was preregistered.
